# Genotyping human ancient mtDNA control and coding region polymorphisms with a multiplexed Single-Base-Extension assay: the singular maternal history of the Tyrolean Iceman

**DOI:** 10.1186/1471-2156-10-29

**Published:** 2009-06-19

**Authors:** Phillip Endicott, Juan J Sanchez, Irene Pichler, Paul Brotherton, Jerome Brooks, Eduard Egarter-Vigl, Alan Cooper, Peter Pramstaller

**Affiliations:** 1 Museèe de l'Homme, 17 place du Trocadero, 75116 Paris, France; 2 Instituto Nacional de Toxicología y Ciencias Forenses, Campus de Ciencas de la Salud,38320 La Laguna, Tenerife, Spain; 3 Institute of Genetic Medicine, European Academy of Genetics, 39100 Bolzano-Bozen, Italy; 4 Department of Physiology, Anatomy and Genetics, University of Oxford, Oxford, OX1 3PT, UK; 5 Australian Centre for Ancient DNA, University of Adelaide, Adelaide SA 5005, Australia; 6 Department of Pathology, General Regional Hospital, Bolzano-Bozen, Italy; 7 Department of Neurology, Lübeck, Germany

## Abstract

**Background:**

Progress in the field of human ancient DNA studies has been severely restricted due to the myriad sources of potential contamination, and because of the pronounced difficulty in identifying authentic results. Improving the robustness of human aDNA results is a necessary pre-requisite to vigorously testing hypotheses about human evolution in Europe, including possible admixture with Neanderthals. This study approaches the problem of distinguishing between authentic and contaminating sequences from common European mtDNA haplogroups by applying a multiplexed Single-Base-Extension assay, containing both control and coding region sites, to DNA extracted from the Tyrolean Iceman.

**Results:**

The multiplex assay developed for this study was able to confirm that the Iceman's mtDNA belongs to a new European mtDNA clade with a very limited distribution amongst modern data sets. Controlled contamination experiments show that the correct results are returned by the multiplex assay even in the presence of substantial amounts of exogenous DNA. The overall level of discrimination achieved by targeting both control and coding region polymorphisms in a single reaction provides a methodology capable of dealing with most cases of homoplasy prevalent in European haplogroups.

**Conclusion:**

The new genotyping results for the Iceman confirm the extreme fallibility of human aDNA studies in general, even when authenticated by independent replication. The sensitivity and accuracy of the multiplex Single-Base-Extension methodology forms part of an emerging suite of alternative techniques for the accurate retrieval of ancient DNA sequences from both anatomically modern humans and Neanderthals. The contamination of laboratories remains a pressing concern in aDNA studies, both in the pre and post-PCR environments, and the adoption of a forensic style assessment of *a priori *risks would significantly improve the credibility of results.

## Background

It is often hoped that the fields of ancient DNA (aDNA) and population genetics will converge to provide novel insights into human prehistory, but there have been very few convincing aDNA studies conducted on human remains, due to the difficulty of differentiating between authentic results and contaminating DNA [1-4]. This problem is particularly acute when the putative endogenous sequences are similar to possible contaminants, and for this reason the most credible research has been restricted to the investigation of remains with highly distinctive, individual or population-specific, genetic signatures, e.g. [5-8]. Consequently, a number of high-profile human aDNA studies have been questioned on the grounds of insufficient proof of authenticity [1,9-12].

Traditionally, the molecular tool for the identification of both endogenous human DNA and potential contaminant sequences has been the comparatively high levels of polymorphism found in the first and second hypervariable sections (HVS1, HVS2) of the mitochondrial (mtDNA) control region. The rapid rate of evolution in this short stretch of the molecule increases the likelihood of encountering multiple distinctive single nucleotide polymorphisms (SNPs) in single overlapping polymerase chain reaction (PCR) products. However, in practice, the utility of the control region for this purpose is often compromised by elevated levels of homoplasy, where identical sequences can be shared between different haplotypes. This has lead to an increasing use of hierarchical coding region SNPs to better discriminate between separate haplotypes with identical HVS1 sequences [13]. Whilst this is relatively unproblematic for modern DNA samples, in the case of ancient samples, such as the Iceman, the production of control and coding region sequences in separate PCRs makes it difficult to unequivocally rule out contamination as the source of individual results [12,14,15]. So, paradoxically, the most credible examples of this approach are those where the control region motif is already distinctive in its own right, and the provision of a single additional SNP often provides no significant additional resolution, as with the five Amerindian mtDNA haplogroups, e.g. [16,17].

The most critical factor affecting the accurate genotyping of mtDNA data comprised of both SNPs and control region sequences is the reliability of interpreting unlinked PCR products. These may be subject to sporadic contamination and differential amplification of endogenous and contaminant DNA. One way to address this issue for SNPs is to simultaneously characterise multiple polymorphic sites in a two-stage multiplex reaction using a linear Single-Base-Extension (SBE) mini-sequencing reaction [18-22]. These can overcome many of the problems inherent in providing phylogenetic resolution for human aDNA samples and, in principle, may be able to differentiate between endogenous DNA and a possible contaminant from the same mtDNA haplogroup. Here, we extend this principle to genotype both control region polymorphisms and hierarchical coding regions SNPs within a single multiplexed SBE reaction (K-plex). This is used to obtain a detailed genotype of the Tyrolean Iceman's mtDNA using three separate extracts made from samples of bone and faecal material. The robustness of the results are explored through controlled contamination experiments using SBE reactions and quantitative PCR, and by providing an evaluation of the *a priori *possibility of obtaining an identical genotype from contaminants originating within the laboratory. The discussion includes a detailed reanalysis of the original study of the Iceman's mtDNA [14] and the conclusions serve to highlight the problems of achieving authentic results from European aDNA samples in general, and the fallibility of independent replication, in particular.

## Results

### Cloned Sequences

**Figure 1:**
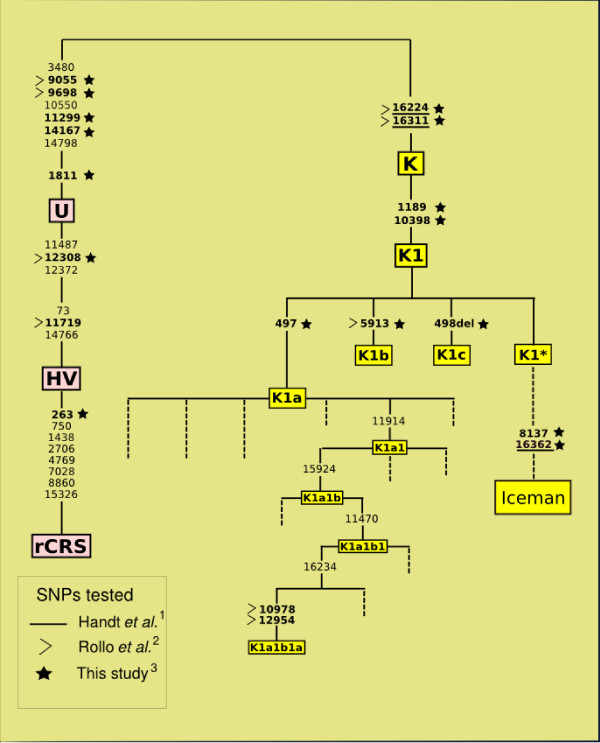
**PCR results for the three extracts of the Iceman's DNA (HB49, HB50, BPE1)**. The results place the Iceman's mtDNA into a new (undefined) phylogenetic position (K1*) whilst also reporting the transitions at nps 8137 and 16362. Human mtDNA mutations are scored relative to the revised Cambridge Reference Sequence (rCRS) [23,24], so the Tyrolean Iceman's mtDNA is 'ancestral' at the three positions (C497T, G5913A, 498delC) that define K1a, K1b, and K1c. The sites tested in, ^1 ^[14], ^2 ^[15] and ^3^this study, are shown to compare the phylogenetic discrimination achieved. Critically, the 16362C polymorphism was not reported in the original study [14] and not tested for in [15]. A recent study [25] explored the entire mtDNA genome of the Iceman by singleplex PCR and has reported all of the polymorphisms listed in Table 1.

The results from initial PCR amplification and direct sequencing of the three new DNA extracts are reported in Figure 1 and Table 1 and characterise the Iceman's diagnostic control region polymorphisms. The first hyper-variable section (HVS1) between nucleotide positions (nps) 16040 and 16401, revealed three consistent polymorphisms (16224C-16311C-16362C), relative to the revised Cambridge Reference Sequence (rCRS) [23,24], in all clones from all samples (see additional file 1 showing clones covering the key sites nps 16311 and 16362 from all three extracts). There were no positive results obtained from the corresponding extraction blanks and negative controls used in each amplification. The results suggest that the mtDNA belongs to K1* by virtue of matching the revised Cambridge Reference Sequence (rCRS) [24] at nps 497 (K1a), 498 (K1c) and 5913 (K1b), whilst being derived at nps 1189 and 10398 (both K1). Importantly, the presence of the transition at np 8137 is unique within contemporary hg K [13], and the mutation at np 16362 was not reported in the original study of the Iceman's mtDNA [14].

**Table 1:** Singleplex PCR results for the bone (BPE1, HB50) and faecal (HB49) extracts of the Iceman's mtDNA, showing all polymorphisms obtained in separate sets of cloned sequences.

	Control Region						Coding Region										**P1**	**P2**
			
**HB50**	**G**263G	16224C	16311C	16362C	497T	498delC	1189C	1811G	5193A	8137T	9055A	9698C	9716C	9962A	10398G	14167T	**00100**	**00207**
**HB50**																	**00157**	**00290**

**HB50**																	**16015**	**16179**

**HB50**																	**16074**	**16231**

**HB49**		**C**															**16131**	**16300**

**HB50**		**C**	**C**	**C**													**16186**	**16398**

**BPE1**		**C**	**C**	**C**													**16192**	**16402**

**HB49**			**C**	**C**													**16257**	**16398**

**HB50**		**C**															**16186**	**16291**

**HB50**			**C**														**16257**	**16363**

**HB50**				**C**													**16320**	**16424**

**HB50**					C	C											**00460**	**00524**

**HB50**							**C**										**01142**	**01220**

**HB49**								**G**									**01781**	**01848**

**HB50**								**G**									**01781**	**01848**

**HB50**									G								**05846**	**05950**

**HB50**										**T**							**08105**	**08205**

**HB49**										**T**							**08105**	**08205**

**BPE1**										**T**							**08105**	**08205**

**BPE1**											**A**						**09029**	**09095**

**HB50**												**C**	T				**09636**	**09742**

**HB50**														G			**09922**	**10005**

**HB50**															**G**		**10353**	**10442**

**HB49**																**T**	**14105**	**14207**

**Con.**	**G**	**C**	**C**	**C**	C	C	**C**	**G**	G	**T**	**A**	**C**	T	G	**G**	**T**		
		
**rCRS**	A	T	T	T	C	C	T	A	G	C	G	T	T	G	A	C		

In this table alleles derived from the revised reference sequence (rCRS) are shown in bold and with gray background. The consensus (Con.) is given above the Cambridge Reference Sequence (rCRS) [23,24] in the last but one row. The alleles 263G, 1189C, 1811G, 9055A, 9698C, 14167T, 16224C and 16311C are typical of haplogroup (hg) K1, lying within macro-haplogroup R of the human mtDNA genealogy [13]. The Iceman's mtDNA is further characterised by 8137T (so far unique within hg K) and 16362C. The last two columns (P1, P2) give the 5 prime position of each primer used for the amplification, full details of which are in additional file 4.

### K1 SBE multiplex

Due to the difficulty inherent in proving that results matching the reference sequence at nps 497, 498 and 5913 were not due to contamination [12], a multiplex SBE assay (K1-plex) was designed to test for these three HVS1 polymorphisms (16224C-16311C-16362C), plus nine hierarchical SNPs from both the control and coding regions. The multiplex results revealed transitions at nps 1189, 1811, 9698, 10398 and 14167 (Table 2), indicating membership of the human mtDNA sub-haplogroup K1 [13], as previously suggested by [15,25]. In addition, because the Iceman's sequence matches the rCRS at nps 497, 498, and 5913 (diagnostic of K1a, K1c, and K1b, respectively), we can extend previous suggestions that the Iceman's genotype does not belong to any of the three known clades of K1, and should be referred to a new (undefined) paraphyletic clade, of K1 [25] (Figure 1). A transition at np 8137, previously discovered by singleplex assay of the Iceman's mtDNA, was confirmed by the multiplex SBE results, and is currently unique within hg K [13]. All multiplex results were cross-checked by cloned single-plex PCR results to investigate the potential for false positives in the SBE reaction. The allelic state of each of the 11 SNPs matched the multiplex results (details of all singleplexes are given in Table 1).

**Table 2:** Multiplex Single Base Extension results for the HB50 bone extracts of the Iceman's DNA using the K1 multiplex.

**Sample**												
**rCRS**	MP	T	T	T	C	C	T	G	C	T	A	T

**HB50**	**K1**	**C 16224C**	**C 16311C**	**C 16362C**	C**497T**	C**498 elC**	**C1189C**	G**5913A**	**T8137T**	**C11299C**	**G12308G**	**C12705C**

**Figure 2:**
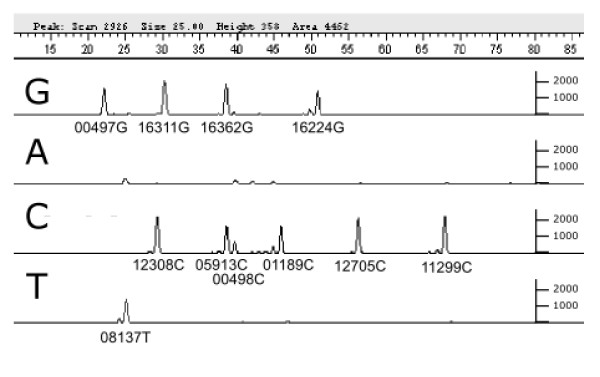
**Representative electropherogram of the K1 multiplex assay conducted on the HB50 aDNA extract made from the Iceman's bone, reporting all three HVS1 mutations revealed in the singleplex assays (16224–16311–16362) and eight additional phylogenetically informative SNPs**. The SBE results are differentiated by a combination of the alleles and their electrophoretic mobility, determined by the length of the primers (see Table S4). All alleles are called relative to the strand of origin targeted in the SBE reaction; those from the reverse strand should be complemented to be consistent with the rCRS (see Table S2). Positions 5913 and 498 (the smaller peak of the two) have coalesced slightly due to departures from the predicted electrophoretic mobility of the SBE primers. Minor peaks to the left and immediately adjacent of some SNP positions represent background noise, due to variation in length of some SBE primers and can be ignored. There is some background noise evident on the A system but only the peak at the 16362 position is in the correct position to represent contamination (see Fig. 3).

**Figure 3:**
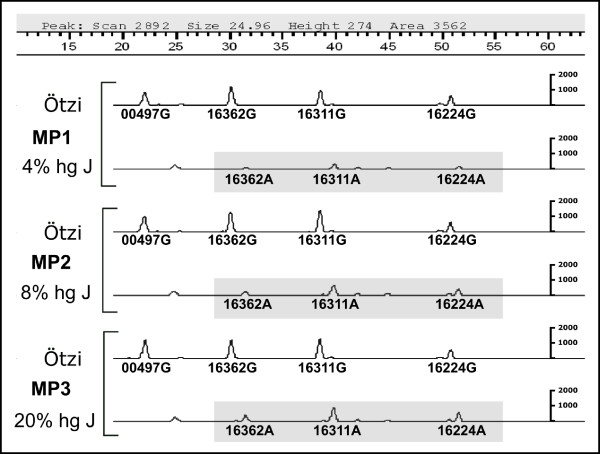
**Results of the contamination assay showing the G/A system from three different multiplex SBE reactions (MP1, MP2, MP3), initiated with the Iceman's DNA mixed with three different amounts of an hg J contaminant**. Because the contaminant has ancestral alleles at nps 16224, 16311 and 16362, this causes additional peaks to appear in the A system (highlighted). The small peak to the left of 16362A in each case is background noise (i.e. not due to primer length variation which would produce multiple peaks of varying size, see the G system in Figure 2. This does not interfere with the base calling because it is outside the range of either the 00497 or 16362 SBE primers. The sensitivity of this method is demonstrated by its ability to detect the second population of templates (hg J) at the level of ~4%; even at levels of ~20% contamination, the results for the Iceman's mtDNA are unambiguous and unaffected by the contaminant. The ability to test multiple sites in a single reaction, in the presence of contaminants, without the need to clone individual PCR products, represents a significant advantage of the SBE methodology.

For the sake of consistency with Table 1, alleles read from the forward DNA strand, relative to the revised Cambridge Reference Sequence (rCRS) [23,24], unlike Figures 2 and 3 that show the state according to the actual strand targeted by the SBE assay. These results are identical to the cloned singleplex results shown in Table 1, demonstrating consistent genotyping of the Iceman by different methods. As all these results were obtained in a single multiplex reaction, credibility is given to those reactions returning the rCRS. Such results in a singleplex scenario could be explained by contamination of an individual assay by any non hg K mtDNA source.

Crucially, the actual SBE traces (Figure 2) show that the results originated from a single effective population of DNA templates because there were no additional alleles at any of the sites tested. The use of multiplexed SBE also ensured that those hg K1 diagnostic sites matching the rCRS (nps 497, 498, 5913) were not a result of contamination, or sample mix-up, because all were typed concurrently in a single assay, together with other SNPs (16362C, 8137T) diagnostic of the Iceman's mtDNA. Real-time PCR quantification (see additional file 2) indicated that the multiplex assay was initiated with the equivalent of ~36,000 molecules of mtDNA capable of amplifying 60 bp and that inhibition was not detected. This effective copy number is consistent with the absence of repetitive damage motifs amongst the clones and the lack of allelic drop-out in the SBE results [26].

### Contamination controls

To further test the potential effect of contamination on the results, a second K1 multiplex assay was performed with the addition of various quantities of a second DNA extract (hg J) to imitate the role of modern contaminant. This produced clearly observable double peaks (representing different alleles at the same site) across a large range of dilutions (Figure 3), confirming the presence of two populations of templates. The lowest quantity of contaminant detected by the multiplex assay was measured by real-time quantitative PCR to be equivalent to ~4% of the total starting copy number. As the proportion of the contaminant increases, so do the secondary peaks, but even at levels of ~20% it is still possible to unambiguously distinguish the two separate populations of templates. This result strongly supports the conclusion from the cloned HVS1 sequences (Figure S1) that the same single endogenous population of templates is represented in the three extracts of the Iceman's DNA.

We also typed the mtDNA of all workers involved in the study, from the removal of the samples from the mummy in Italy to the extraction of the DNA in Oxford and no individual had mtDNA belonging to hg K. Although positive results from extraction blanks and PCR negative controls did not occur during the present study, a human contaminant was identified amongst earlier work conducted in the Oxford isolation facility. This was from a modern source of hg K mtDNA with the control region motif 16224C-16311C (i.e. without 16362C) and was eliminated by modification to laboratory protocols regarding common consumables (see Methods).

This hg K contaminant, together with all aDNA extracts with putative European mtDNA prepared in the isolation facility, is listed in additional file 3 but these sequences include no examples of hg K with 16362C. Therefore, at the time that the research into the Iceman's mtDNA was conducted, the probability of obtaining a matching hg K contaminant in the Henry Wellcome Ancient Biomolecules Centre appears to have been extremely low.

## Discussion

### The significance and authenticity of the transition T16362C

Revisiting the mtDNA of the Tyrolean Iceman has raised the issue of how to differentiate between conflicting but highly similar sets of aDNA results, in this case from independent studies of the same individual [[14,15,25], this study]. Although each study obtained hg K mtDNA in their PCR products, only two reported the 16362C polymorphism [[25], this study]. Therefore, there would appear to be multiple sources of hg K mtDNA templates involved because of this difference in the control region motifs. It is important to note that this single base change (T>C) cannot derive from post-mortem damage to the mtDNA molecule because this results in C>T changes only, from the deamination of cytosine to uracil, which is read as a thymine, and appears as a G>A on the opposite strand [27]. Therefore, an erroneous T>C transition could only derive from an artefact of PCR, but the chances of this occurring in multiple amplifications from our three samples, and for the same artefact becoming fixed in every PCR product, appear to be remote. The lack of variation at all the sites we tested, both in cloned sequences and SBE assays, is only consistent with the presence of a single population of mtDNA templates from all three extracts with the control region motif of 16224C-16311C-16362C, for which the *a priori *probability of these deriving from a common contaminant, either of the laboratory or the sampling procedure, has been demonstrated to be extremely low.

### Phylogenetic evidence of authenticity

**Figure 4:**
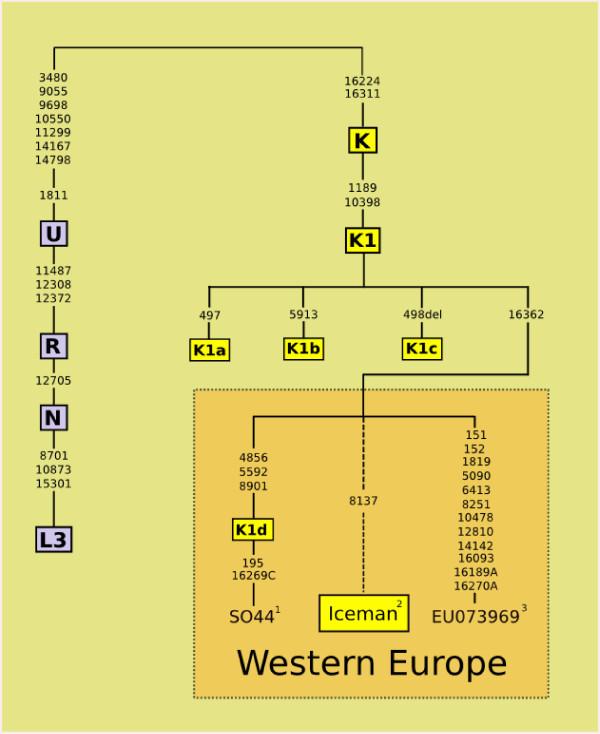
**Phylogenetic tree of the main branches of human mtDNA hg K1 showing the western European paraphyletic lineages falling outside of K1a, K1b and K1c**. All mutations are transitions unless indicated by a letter. Dotted lines indicate samples for which replicated whole genome data is not available; SNPs denoted in bold are those tested in the K1 multiplex; the tree is rooted in African hg L3. All lineages with 16362C are grouped by parsimony into a single potential European paraphyletic clade. The clade of K1d is defined by three coding region mutations shared between SO44 and Oc5 from [29]. Together with a single example of K1* from Iran [13] (not shown), these represent the sum total of hg K1 mtDNA lineages currently known to fall outside of K1a\K1b\K1c, amongst a worldwide survey of over 1000 haplogroup K samples (^1^Howell *et al*., 2003 [28]; ^2^this study; ^3 ^Genbank direct submission).

The likelihood of the current results representing the endogenous DNA of the Iceman is substantially increased by the haplotype falling outside of K1a/K1b/K1c, combined with the unique hg K transition C8137T, matching the results of [25]. A Genbank search reveals two contemporary coding region genomes (SO44 from [28] and Oc05 from [29]) that can be securely assigned to the same paraphyletic K1* position as the Iceman and which share three coding region polymorphisms (see Figure 4). Only one, a Dutch sample (SO44 from [28]) has a published control region sequence, but this also displays the T16362C transition. We propose that these two genomes define a new clade K1d defined by the coding region motif of 4856C-5592G-8901G. This finding provides some support for the proposal of a single European paraphyletic clade of mtDNA hg K1 defined by the 16362C polymorphism [25]. However, the use of a single mutational hotspot (i.e. np 16362, which occurs at least eight times on the hg K background alone) for this purpose is usually discouraged [30]. If all these genomes belong to a single clade, then convention suggests that it should be named K1d, following the cladistic rules for the hierarchical ordering of haplogroups and sub-haplogroups formalised by [31].

### The perils of contamination

**Figure 5:**
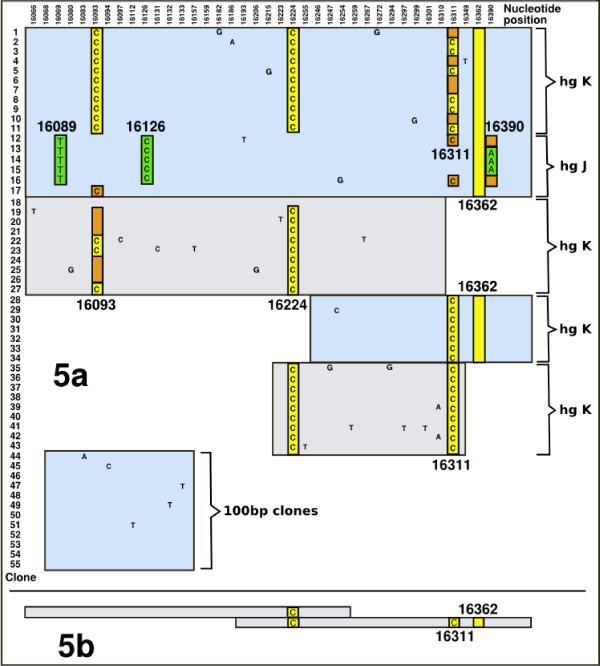
**a/b Graphical representation of the sequences obtained for the Iceman from Handt et al. 1994**. **(a) **Cloned HVS1 control region sequences from [14] (the nucleotide positions are listed in the top row, whilst the first column refers to the number of each clone). There are two clearly identifiable European mtDNA haplotypes belonging to hg K (16093C-16224C-16311C, clones 1–11 and potentially 19–43), and hg J (16069T-16126C-16390A, clones 12–16). The absence of both 16093C and 16126C from the shortest (100 bp) amplification product (clones 44–55) indicates the presence of at least three populations of templates. Positions of template switching ('jumping PCR') are indicated by brown highlighting, and evident by the replacement of the expected mutation either by the rCRS (no letter), or a polymorphism from a different template. Critically, there are no examples of the 16362C polymorphism in any templates covering this position. **(b) **Results from the independent replication [37] which were only indirectly sequenced (not cloned), identifying just the two control region transitions basal to hg K (16224C-16311C). This suggests that these results derived from a second hg K contaminant, differing from those of Figure 5a through the lack of the 16093C transition, and from the Tyrolean Iceman by the absence of the key T>C transition at np 16362.

The difference between the conclusions of the original study and the current consensus of the Iceman's mtDNA control region might appear to be trivial. Yet the implications are actually of the utmost importance and warrant a detailed reanalysis of the original data. This is because the absence of the 16362C allele amongst the results of Handt *et al *[14], suggests that they genotyped a different hg K individual. Reanalysis of the original data reveals the presence of at least two populations of templates (Figure 5a). One of these is a variant of human mtDNA hg J (defined by the motif 16069T-16126C-16390A) [21,32], whilst the other observable haplotype (16093C-16224C-16311C) is a common occurrence within hg K [13].

### Appropriate Molecular Behaviour

The published consensus sequence excluded the transition detected at np 16093 on the basis that it did not feature in the 100 bp product [14]. This short sequence was argued to have been more likely to represent the endogenous DNA on the basis of appropriate molecular behaviour [14]. This concept predicts that the shortest amplification product, in the presence of modern contaminants, is more likely to represent the endogenous DNA [33,34] (but see also [35]). As we have demonstrated that the mtDNA of our Iceman extracts does not contain the 16093C allele, it is conceivable that the 100 bp product of Handt *et al*. [14] did originate from DNA endogenous to the sample. Support for, or against, this conclusion can be sought from the clones for sequences including np 16362. It was recently suggested that the lack of the 16362C allele amongst the Handt *et al*. sequences may partly be attributable to the production of chimeras amongst these longer amplicons [25]. The obvious mixture of alleles at nps 16093, 16311 and 16390 (clones 1, 4, 6, 7, 10, 12, 16, 17, 19–21, and 24–26; Figure 5a) demonstrate that either these sites were subject to post-mortem damage or hg K mtDNA templates were involved in 'jumping PCR' events with contaminants [36]. Yet, of all the clones covering the upper portion of the control region sequence (1–17, and 28–34), none contain the key transition at np 16362. This suggests that the extraction actually contained a different source of hg K mtDNA to the single mtDNA sequence recovered in [25] and the present study.

### The fallibility of independent replication

Given the absence of 16093C and 16362C amongst the replication results of Handt *et al*., the only plausible conclusion is that these derived from a second, entirely different, hg K source. These results, combined with a unique haplotype in [25] and the present study, strongly suggests that *both *laboratories in the original study suffered from hg K contamination. The mtDNA profiles of the staff in the primary laboratory of the first study were not provided [14]; although a subsequent publication of the replicated results [37] disclosed that a member of the secondary laboratory staff was hg K (16224C-16311C), substantially increasing the likelihood for contamination occurring. It is important to note that the independent replication step, in this case, did not technically fail. Instead, the analysis did not manage to distinguish between these two non-identical sources of hg K mtDNA, neither of which appear to have derived from the Iceman. The fallibility of the original, independently replicated, study emphasizes why unlinked singleplex PCR results may not be sufficient proof of authenticity. This is particularly problematic where novel aDNA genomes are defined by long stretches of sequence matching the rCRS, and motifs including C>T changes that can arise as a product of post-mortem modifications to DNA templates [25].

### Recommendations for future human aDNA research

#### Criteria of authenticity

We suggest that any contamination risk assessment in human aDNA research would be improved through the incorporation of an analysis of the *a priori *probability that the extract will be contaminated. Human aDNA studies are now routinely providing genetic characterisation of individuals who have been involved in the manipulation of a sample, e.g. [17]. For an accurate evaluation of the true risk, in a way that would satisfy common international standards in forensic science, it is also necessary to supply data on false positives and other previous amplification of similar genotypes from the laboratories concerned. The presence of hg K mtDNA amongst the common reagents of the laboratory in the present study confirms how consumables can be a source of contamination [38], with obvious implications for the efficacy of independent replication. The findings of the present study provide clear evidence why, as with any scientific investigation, all reasonable efforts to falsify human aDNA results should be made, even when controls such as independent replication are known to be fallible under certain circumstances [39]. This is clearly the case for human aDNA research carried out on European remains in European laboratories.

#### Identifying DNA haplotypes using multiple SNPs

The use of the K1-plex demonstrates the potential power of discrimination achievable by linking control and coding region sites in a single SBE multiplex. Although the fortuitous circumstances of a unique maternal lineage cannot be expected in the majority of human aDNA studies, this kind of multiplexed approach should in many situations be able to identify where DNA templates from two or more discrete individuals are present. So, the standards applied here should not be taken as a reference for other research using human DNA extracts that fall squarely into more mainstream situations. Nevertheless, the linking of all loci in one multiplex SBE reaction can still improve the security of interpretation, whilst assisting in the identification of possible contamination events and helping to conserve irreplaceable aDNA extracts.

## Conclusion

This study has highlighted the potential fallibility of the interpretation of human aDNA results, and the need for a forensic style assessment of risks associated with the production of data. Independent replication remains a core component of authentication strategies for human aDNA studies, and it is paramount that this test is applied critically to function properly. More stringent controls are required to move the field forward, both *a priori *and *a posteriori*, for the production and interpretation of data; in particular, the disclosure of all possible sources of contamination in aDNA studies using generic hominin primers. In PCR-based studies, this data should include genetic profiles of the workers involved for whichever target loci are being investigated.

The use of multiplex SBE assays offer one solution for authenticating human aDNA results and screening novel genome data. In the present study the K1 multiplex accurately genotyped the Iceman's mtDNA and has independently confirmed it belongs to a novel lineage of mtDNA hg K. A novel aspect of this approach is the ability to link SNPs from both the control and coding regions of the mtDNA genome in a single reaction, reducing the possibility of contamination affecting the results. In a controlled contamination experiment, the SBE methodology correctly identified the endogenous genotype of the Iceman and the relative quantities of the modern mtDNA contaminant.

## Methods

Two samples of bone removed from the ileum, and one of faecal matter from the rectum were digested in temporally separated extractions (4 ml total volume) by a previously published protocol [22]. One extraction blank was produced for each sample, and PCRs also included negative controls.

The specialist Ancient Biomolecules Centre (ABC) at Oxford was used to extract DNA, and set up PCR reactions. The ABC is physically isolated, subject to stringent anti-contamination procedures, equipped with positive air pressure and UV lighting, and has a DNA laboratory and equipment (glove box, instruments, full body suits, protective masks, etc.) dedicated solely to ancient human specimens. The thermal cycling reactions and subsequent downstream work took place in a separate laboratory located in the Department of Zoology.

Initial singleplex PCRs on the control region were conducted with primers listed in additional file 4 and cloned by previously published protocols [22]. Several hierarchical SNPs from the human mtDNA phylogeny together with haplogroup K specific SNPs were identified [13,40] using additional singleplex PCR primers designed to be compatible under multiplex PCR conditions [26].

The three control region SNPs, located in the singleplex PCRs, were combined with key SNPs from major clades of the hg K phylogeny, to form the K1 multiplex. A selection of higher order hierarchical SNPs from the human phylogeny were also included to check the consistency of results across additional loci [13,40]. This integration of all diagnostic sites into a single assay reduces the range of explanations required when results do not conform to expectations because the possibility of sample mix up is eliminated.

The initial multiplex PCR was performed using 2 *μ*l of DNA extract in a 25 *μ*l reaction volume using primers listed in additional file 5 by a previously published protocol [22]. SBE reactions were performed in a total volume of 5 *μ*l with 0.5 *μ*l purified PCR product, 2.5 *μ*l SNaPshot™ reaction mix (AB) and 0.5 *μ*l SBE primer mix (SBE primers listed in additional file 6). Negative controls were included in both stages of the assay to assess the potential for contamination.

Automated allele calls were made using macros constructed in Genotyper 3.7 (AB). Primer concentrations were adjusted to give balanced amounts of products and peak heights, concentrating on the SBE reaction [19]. All primers were synthesized and reverse-phase HPLC purified by Biomers.net GmbH (Ulm, Germany). A detailed protocol for the optimization of SBE multiplexes is available elsewhere [26].

Quantitative PCR (qPCR) was conducted on the HB50 aDNA bone extract and a modern DNA sample (belonging to the mitochondrial hg J) in 25 *μ*l reaction volumes, using SYBR Green PCR master-mix (AB), 300 nM human-specific primers, synthesized template standards (see additional file 7) and an ABI Prism 7000 Sequence Detection System (95°C for 10 min, followed by 40 cycles of 95°C for 15 sec, and 60°C for 60 sec). All samples were run in triplicate with two 1:4 serial dilutions. The standards were also run in triplicate with 1:9 dilutions ranging from 10^6^-10^2 ^copies per *μ*l. The adjusted R^2 ^value of all assays performed was equal to a minimum of 99%, and PCR efficiency, in each case, was calculated with the formula [41] to be ~2 (see additional file 2).

Once the effective copy number of the modern DNA was known, a range of dilutions were run against the HB50 extract in a relative quantification, using the same primers and conditions, until the reported copy number of both the ancient and modern extracts were equivalent. The multiplex contamination experiments were then performed using successive serial dilutions of the hg J contaminant mixed with constant quantities of the aDNA extract to seed the multiplex PCR. This procedure provides a detailed measure of the sensitivity of the SBE methodology for identifying and estimating the amount of contamination present in an aDNA extract through the incorporation of additional peaks at segregating sites.

All contamination experiments, thermal cycling reactions and downstream work took place in a separate laboratory located in the Department of Zoology. All relevant personnel in this study had their mtDNA typed. The two researchers who took the samples from the Iceman have mtDNA matching hg H and hg J1b. All aDNA work in Oxford (extractions, singleplex and multiplex PCRs) were carried out by PE, who is hg H.

Figures were prepared using Inkscape Vector Illustrator compiled for use in Ubuntu Linux. The real-time PCR plot was created using the R statistical package . The manuscript was written using the LyX document preparation system .

## Authors' contributions

PP and AC were responsible for the original idea of the study. PE conceived, designed and performed the experiments; analysed and interpreted the data; drafted the manuscript. JJS participated in the design of the study; designed the multiplex, and helped to draft the manuscript. IP performed analysis of modern DNA samples and edited the manuscript. PB helped with the design of the study and helped to draft the manuscript. JB helped to design and run the real-time PCR quantitation and helped to draft the manuscript. EV supplied the samples. All authors read and approved the final manuscript.

## Supplementary Material

Additional file 1 **Cloned HVS1 sequences from the Iceman**. HVR1 sequences covering diagnostic SNPs at nps 16311 and 16362, obtained from three extracts of the Iceman's DNA (BPE1, HB49, HB50). Click here for file

Additional file 2 **qPCR results for the Iceman (extract HB50)**. The blue diamonds are the various dilutions of the reference standard, and the red ones represent the Iceman extract. This gives the estimated starting copy number per microlitre of extract (~18 k). Click here for file

Additional file 3 **Table of mtDNA haplotypes**. List of mtDNA haplotypes of all results from aDNA extracts an negative controls (NC) with putative European human mtDNA prepared in the ABC isolation facility in Oxford. rCRS means that there were no mutations observed relative to the revised Cambridge Reference Sequence. Click here for file

Additional file 4 **Table of Singleplex PCR primers used in this study**. The first and fourth columns give the 5' ends of the forward and reverse primers, respectively. Click here for file

Additional file 5 **K1 multiplex PCR primers**. The fourth and the sixth columns give the 5' ends of the forward and reverse primers, respectively, whilst the second column indicates the position of the site being targeted in the SBE reaction. Click here for file

Additional file 6 **K1 multiplex SBE primers**. The name of the Single-Base-Extension primer indicates the site being tested and the strand orientation, whilst the last column gives the possible ancestral and derived alleles. Click here for file

Additional file 7 **Quantitative PCR primers and synthetic standards**. The name of the primer indicates the position immediately 3' of the primer sequence. Click here for file
