# Supplementary material for: Genotyping human ancient mtDNA control and coding region polymorphisms with a multiplexed Single-Base-Extension assay: the singular maternal history of the Tyrolean Iceman
Source: BMC Genet. 2009 Jun 19;10:29. doi: 10.1186/1471-2156-10-29 (PMC2717998; doi:10.1186/1471-2156-10-29)
Supplement: Additional file 3 — Table of mtDNA haplotypes. List of mtDNA haplotypes of all results from aDNA extracts an negative controls (NC) with putative European human mtDNA prepared in the ABC isolation facility in Oxford. rCRS means that there were no mutations observed relative to the revised Cambridge Reference Sequence. [file 1471-2156-10-29-S3.pdf]

Extract No.	Sample	Region	Material	Age	Origin	PCR Date	16204_16339	Possible ID
HB25	Grob13	Europe	Bone	Neolithic	Mainz	01/06/05	rCRS	rCRS
HB26	SZA 23 3d	Europe	Bone	Neolithic	Mainz	01/06/05	rCRS	rCRS
HB38	SFO.CG R4	Mallorca	Bone	Neolithic	Private	01/10/05	255Y, 278Y	hg X
HB39	SFO.CG R5	Mallorca	Bone	Neolithic	Private	01/10/05	rCRS	rCRS
HB40	SFO.CG L2	Mallorca	Bone	Neolithic	Private	01/10/05	353Y	Damage
HB60	Don 1C	Europe	Bone	Neolithic	Mainz	01/06/05	264Y	Damage
HB61	SZA 23 3d	Europe	Bone	Neolithic	Mainz	01/06/05	254A	hg H
HB62	Grob 13	Europe	Bone	Neolithic	Mainz	01/06/05	rCRS	rCRS
PE232	K78h	Samations	Tooth	Neolithic	Mainz	01/06/05	rCRS	rCRS
PE234	K8n3	Samations	Tooth	Neolithic	Mainz	01/06/05	223T, 235T	hg X
NC	H20	na	na	na	na	20/10/04	235T, 291T	hg H
NC	H20	na	na	na	na	20/10/04	222T, 261T	hg J1b
PEH14	1949.12.7.5	Sri Lanka	Hair	19th Century	NHM London	18/08/04	224C , 311C	hg K
PEH16	1949.12.7.8	Sri Lanka	Hair	19th Century	NHM London	18/08/04	224C , 311C	hg K
PEH18	EB	na	na	na	na	18/08/04	224C , 311C	hg K
NC	H20	na	na	na	na	18/08/04	224C , 311C	hg K
