# Supplementary material for: Genotyping human ancient mtDNA control and coding region polymorphisms with a multiplexed Single-Base-Extension assay: the singular maternal history of the Tyrolean Iceman
Source: BMC Genet. 2009 Jun 19;10:29. doi: 10.1186/1471-2156-10-29 (PMC2717998; doi:10.1186/1471-2156-10-29)
Supplement: Additional file 4 — Table of Singleplex PCR primers used in this study. The first and fourth columns give the 5' ends of the forward and reverse primers, respectively. [file 1471-2156-10-29-S4.pdf]

Primer 1	PCR forward (L) primers	Primer 2	PCR reverse (H) primers	Amplicon
00100	ggagccggagcaccctat	00207	cacactttagtaagtatgttcgcctgt	108
00157	tttatcgcacctacgttcaatattac	00290	tttttgttatgatgtctgtgtgga	134
16015	tattctctgttctttcatggggaag	16179	gatgtggattgggtttttatgtact	165
16074	atcaacaaccgctatgtatttcgta	16231	tagttgagggttgattgctgtactt	158
16131	gtaccataaatacttgaccacctgtagta	16300	ttaagggtgggtaggtttgttg	170
16186	ccctccccatgcttacaa	16398	caagggacccctatctgagg	213
16192	ccatgcttacaagcaag	16402	tggtcaagggacccctatct	211
16257	cacccctcacccactagga	16398	caagggacccctatctgagg	142
16186	ccctccccatgcttacaa	16291	gtgggtaggtttgttggtatccta	106
16257	cacccctcacccactagga	16363	gacgagaagggatttgactgtaat	107
16320	ccatttaccgtacatagcacattaca	16424	atattgatttcacggaggatggt	105
00460	cttttggcggtatgcacttttaac	00524	gtgtgtgtgtgctgggtagg	65
01142	actacgagccacagcttaaaactc	01220	tatcgattacagaacaggctcctc	79
01781	agtaccgcaagggaaagatga	01848	aaggtataggggttagtccttgcta	68
01781	agtaccgcaagggaaagatga	01848	aaggtataggggttagtccttgcta	68
05846	cctgtctttagatttacagtccaatgc	05950	ccaatgtctttgtggtttgtagag	105
08105	gcaattcccggacgtctaa	08205	atgaaactgtggtttgctccac	101
08105	gcaattcccggacgtctaa	08205	atgaaactgtggtttgctccac	101
08105	gcaattcccggacgtctaa	08205	atgaaactgtggtttgctccac	101
09029	acctactcatgcacctaattgga	09095	agtgtagagggaaggttaatggttg	67
09636	atcacctgagctcaccatagtctaa	09742	gaggcttgtaggagggtaaaataga	107
09922	cctgatactggcattttgtagatg	10005	ttatactaaaagagtaagaccctcatcaatag	84
10353	aagtctggcctatgagtgactacaa	10442	tgagtcgaaatcattcgttttg	90
14105	ctttcttcttcccactcatcctaa	14207	ctggatgaacattgtttgttgg	103
