# Supplementary material for: Genotyping human ancient mtDNA control and coding region polymorphisms with a multiplexed Single-Base-Extension assay: the singular maternal history of the Tyrolean Iceman
Source: BMC Genet. 2009 Jun 19;10:29. doi: 10.1186/1471-2156-10-29 (PMC2717998; doi:10.1186/1471-2156-10-29)
Supplement: Additional file 5 — K1 multiplex PCR primers. The fourth and the sixth columns give the 5' ends of the forward and reverse primers, respectively, whilst the second column indicates the position of the site being targeted in the SBE reaction. [file 1471-2156-10-29-S5.pdf]

Assay	Site	𝕃 primer	PCR forward (L) primers	H primer	PCR reverse (H) primers	Size
K1	497/498del	𝕃00460	cttttggcggtatgcacttttaac	H00536	gtgtgtgtgctgggtagg	77
K1	1189	𝕃01142	actacgagccacagcttaaaactc	H01220	tatcgattacagaacaggctcctc	67
K1	5913	𝕃05846	cctgtctttagatttacagtccaatgc	H05950	ccaatgtctttgtggtttgtagag	118
K1	8137	𝕃08105	gcaattcccggacgtctaa	H08205	atgaaactgtggtttgctccac	94
K1	11299	𝕃11266	caccctaggctcactaaacattcta	H11335	gttggctcaggagtttgatagttct	70
K1	12308	𝕃12257	ctttctcaacttttaaaggataacagc	H12336	ttacttttatttggagttgcacca	80
K1	12705	𝕃12663	ctcagacccaaacattaatcagttc	H12766	agccgatgaacagttggaa	104
K1	16224	𝕃16186	ccctccccatgcttacaa	H16291	gtgggtaggtttgttggtatccta	106
K1	16311	𝕃16257	caccсctcacccactagga	H16363	gacgagaagggatttgactgtaat	107
K1	16362	𝕃16320	ccatttaccgtacatagcacattaca	H16424	atattgatttcacggaggatggt	105
