# Supplementary material for: Genotyping human ancient mtDNA control and coding region polymorphisms with a multiplexed Single-Base-Extension assay: the singular maternal history of the Tyrolean Iceman
Source: BMC Genet. 2009 Jun 19;10:29. doi: 10.1186/1471-2156-10-29 (PMC2717998; doi:10.1186/1471-2156-10-29)
Supplement: Additional file 6 — K1 multiplex SBE primers. The name of the Single-Base-Extension primer indicates the site being tested and the strand orientation, whilst the last column gives the possible ancestral and derived alleles. [file 1471-2156-10-29-S6.pdf]

Assay	Primer name	Primer sequence (The added tail is marked in lower case)	Alleles
K1	00497snR	CTGGGTAGGATGGGCG	G/A
K1	08137snF	tGACGTCTAAACCAAACCACTTT	C/T
K1	12308snR	tctTTTTATTTGGAGTTGCACCAAAATT	T/C
K1	16311snR	tctCTATGTACGGTAAATGGCTTTATGT	A/G
K1	16362snR	ctctctctctctctctctctGGGGTCATCCATGGGG	A/G
K1	05913snR	tctctctctctctGTTTGTAGAGAATAGTCAACGGT	C/T
K1	00498DELsnF	ctctctctctctATACTACTAATCTCATCAATACAACCCC	C/G
K1	01189snF	tctctctctctctctctctctctctctCCTGGCGGTGCTTCATA	T/C
K1	16224snR	ctctctctctctctctctctctctGTTGCAGTTGATGTGTGATAGTTG	A/G
K1	12705snF	ctctctctctctctctctctctctctCATTAATCAGTTCTTCAAATATCTACTCAT	C/T
K1	11299snF	tctctctctctctctctctctctctctctctctctctctctctGCTCACTAAACATTCTACTACTCAC	T/C
