# Supplementary material for: Genotyping human ancient mtDNA control and coding region polymorphisms with a multiplexed Single-Base-Extension assay: the singular maternal history of the Tyrolean Iceman
Source: BMC Genet. 2009 Jun 19;10:29. doi: 10.1186/1471-2156-10-29 (PMC2717998; doi:10.1186/1471-2156-10-29)
Supplement: Additional file 7 — Quantitative PCR primers and synthetic standards. The name of the primer indicates the position immediately 3' of the primer sequence. [file 1471-2156-10-29-S7.pdf]

qPCR Primers	Primer name	Primer Sequence
Fwd. Primer	16280F	aacaaacctacccacccttaacagt
Rev. Primer	16340R	tgtgctatgtacggtaaatggcttt
Fwd. Standard	16266F	ccactaggataccaacaaacctacccacccttaacagtacatagtacataaagccatttaccgtacatagcacattacagtcaaatcc
Rev. Standard	16344R	ggatttgactgtaatgtgctatgtacggtaaatggctttatgtactatgtactgttaagggtgggtaggtttgttggtatcctagtgg
